# 987. Clinical Epidemiology and Outcomes of Invasive Pulmonary Aspergillosis as a Complication of Respiratory Viral Infection in Hospitalized Patients

**DOI:** 10.1093/ofid/ofab466.1181

**Published:** 2021-12-04

**Authors:** Bertha A De Los Santos, Brian J Barnes, Nicholas Britt

**Affiliations:** University of Kansas School of Pharmacy, North Kansas City, Missouri

## Abstract

**Background:**

Invasive pulmonary aspergillosis (IPA) is increasingly recognized as a complication of severe respiratory viral infections (RVIs), including influenza and COVID-19. However, the incidence and outcomes of IPA following other RVIs is not well-described. We hypothesized that IPA may be an underreported complication of non-influenza RVIs. The objective of this study was to quantify the incidence and associated outcomes of IPA following RVI in hospitalized patients.

**Methods:**

We conducted a single-center retrospective cohort study of adult hospitalized patients with RVI diagnosed by multiplex PCR-based assay at the University of Kansas Hospital (Kansas City, Kansas) from September 2018-October 2019. Patients with a diagnosis of proven or probable IPA prior to RVI and those with hospital admission < 24 h were excluded from analysis. Proven or probable IPA was defined according to EORTC/MSGERC consensus definitions. The primary outcome was 1-year all-cause mortality.

**Results:**

A total of 195 patients met study criteria and were included in the analysis. The most common types of RVI observed were rhinovirus/enterovirus (57.9%, *n*=113), parainfluenza (13.3%, *n*=26), influenza (8.2%, *n*=16), and respiratory syncytial virus (7.7%, *n*=15). The cumulative incidence of IPA infection within 6 weeks of RVI was 5.6% (*n*=11). Excluding patients co-infected with multiple respiratory viruses (*n*=5), IPA was numerically more likely to occur following influenza compared to non-influenza RVI (12.5% [ *n*=2/16] vs. 4.6% [*n*=8/174]; odds ratio, 2.96; 95% confidence interval [CI], 0.57-15.3; *P*=0.176). Overall, one-year all-cause mortality was 20% (*n*=39/195) in this cohort. Development of IPA as a complication of RVI was associated with a significant decrease in 1-year survival (hazard ratio [HR], 3.04; 95% CI, 1.19-7.78; *P*=0.021), and this relationship persisted after adjustment for age (HR, 2.77; 95% CI, 1.08-7.10; *P*=0.034).

**Conclusion:**

In a cohort of hospitalized patients with RVI, 5.6% of patients developed proven or probable IPA. Although IPA was more likely to occur in patients with influenza, this complication was also observed with other types of RVI. Invasive pulmonary aspergillosis may be an underappreciated complication of non-influenza RVI in hospitalized patients and warrants continued study.

**Disclosures:**

**All Authors**: No reported disclosures

